# Treatment of calvarial defects by resorbable and non-resorbable sonic activated polymer pins and mouldable titanium mesh in two dogs: a case report

**DOI:** 10.1186/s12917-018-1506-0

**Published:** 2018-06-22

**Authors:** Pierre Langer, Cameron Black, Padraig Egan, Noel Fitzpatrick

**Affiliations:** Fitzpatrick Referrals, Orthopaedic and Neurology Hospital, Halfway Lane, Eashing, Godalming, GU7 2QQ UK

**Keywords:** Titanium mesh, Bonewelding®, Calvarial defect, Polymer pins, Bioresorbable, Non-resorbable

## Abstract

**Background:**

To date, calvarial defects in dogs have traditionally been addressed with different types of implants including bone allograft, polymethylmethacrylate and titanium mesh secured with conventional metallic fixation methods. This report describes the use of an absorbable and non absorbable novel polymer fixation method, Bonewelding® technology, in combination with titanium mesh for the repair of calvarial defects in two dogs. The clinical outcomes and comparative complication using resorbable and non-resorbable thermoplastic pins were compared.

**Case presentation:**

This report of two cases documents the repair of a traumatic calvarial fracture in an adult male Greyhound and a cranioplasty following frontal bone tumor resection in an adult female Cavalier King Charles Spaniel with the use of a commercially available titanium mesh secured with an innovative thermoplastic polymer screw system (Bonewelding®). The treatment combination aimed to restore cranial structure, sinus integrity and cosmetic appearance. A mouldable titanium mesh was cut to fit the bone defect of the frontal bone and secured with either resorbable or non-resorbable polymer pins using Bonewelding® technology. Gentamycin-impregnated collagen sponge was used intraoperatively to assist with sealing of the frontal sinuses. Calvarial fracture and post-operative implant positioning were advised using computed tomography. A satisfactory restoration of skull integrity and cosmetic result was achieved, and long term clinical outcome was deemed clinically adequate with good patient quality of life. Postoperative complications including rostral mesh uplift with minor associated clinical signs were encountered when resorbable pins were used. No postoperative complications were experienced in non-resorbable pins at 7 months follow-up, by contrast mesh uplift was noted 3 weeks post-procedure in the case treated using absorbable pins.

**Conclusions:**

The report demonstrates the innovative use of sonic-activated polymer pins (Bonewelding® technology) alongside titanium mesh is a suitable alternative technique for skull defect repair in dogs. The use of Bonewelding® may offer advantages in reduction of surgical time. Further, ultrasonic pin application may be less invasive than alternative metallic fixation and potentially reduces bone trauma. Polymer systems may offer enhanced mesh-bone integration when compared to traditional metallic implants. The use of polymer pins demonstrates initial potential as a fixation method in cranioplasty. Initial findings in a single case comparison indicate a possible advantage in the use of non-absorbable over the absorbable systems to circumvent complications associated with variable polymer degradation, further long term studies with higher patient numbers are required before reliable conclusions can be made.

## Background

Calvarial defects are an unavoidable consequence following resection of primary bone tumors of the skull [1–6] and less frequently following traumatic calvarial fracture [7]. A range of cranioplasty techniques including bone allograft, polymethylmethacrylate (PMMA) and titanium (Ti) mesh have been used to repair skull defects following tumour resection in dogs [1–3, 5]. Calvarial defects of traumatic origin in humans have been treated using customisable mouldable materials including Ti mesh, polyetheretherketone, PMMA and bioceramic hydroxyapatite (HA) [8, 9].

Cranial fractures experience complication due to paucity of available bone stock and limited soft tissue coverage, further complications may arise due to sinus involvement and in such cases care must be given to restore sinusal architecture with internal fixation [10]. Establishing an adequate air-tight seal is of paramount importance in sinus fracture repair [10]. The challenge of repairing irregular bone defects with associated bone-stock loss restricts the use of conventional metal implants in maxillofacial surgery [11]. As an alternative to metallic screws, polymer pins have been used clinically in human craniomaxillary surgery [12]. BoneWelding® technology (WW Technology AG, Schlieren, Switzerland) utilises thermoplastic polymer screws melted by ultrasonic activation, the melted polymer infiltrates surrounding bone geometry, subsequent rapid cooling and hardening providing a comprehensive bone-implant interface [13]. BoneWelding® technology can be applied rapidly with minimal bone trauma and may offer an effective alternative to metallic fixation in calvarial repair.

This case report documents the repair of a traumatic calvarial fracture in a Greyhound and a cranioplasty following frontal bone tumor resection in a Cavalier King Charles Spaniel with the use of a commercially available Ti mesh secured with an innovative thermoplastic polymer screw system. The treatment combination aimed to restore cranial structure, sinus integrity and cosmetic appearance. The clinical application, outcomes and complications are reported and contrasted when using resorbable (case 1) and non-resorbable (case 2) thermoplastic pins. The authors acknowledge that only one case of each system is represented therefore definitive recommendations cannot be made on the suitability of one system versus the other, however the authors do propose that findings described may contribute to clinical decision making in future scenarios.

## Case presentation

### Case 1

A nine-year-old entire male greyhound presented with head trauma resulting from a collision with a park bench. The dog had no previous significant clinical history. On examination, cardiovascular parameters were stable overt distress in the animal not apparent. The dog was ambulatory with normal gait and posture devoid of proprioceptive deficits.

Thorough head inspection revealed subcutaneous emphysema between the eyes and a superficial cut to the right dorso-orbital region. Mild right unilateral epistaxis was noted. The dog resented palpation of the right frontal bone and a communication with the sinonasal cavity was inferred by the presence of a flail segment movement of the bone synchronous with respiration. Cranial nerve examination demonstrated bilateral delayed pupillary light reflex (PLR) and normal pupil size, the remainder of the neurological examination was within normal limits.

**Fig. 1:**
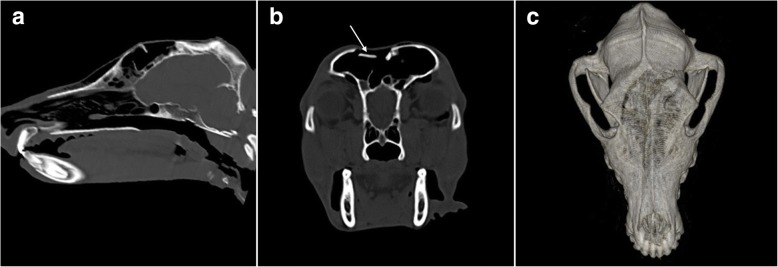
**a** Parasagittal reconstruction of the skull showing the calvarial fracture at the level of the frontal bone. **b** Transverse image at the level of the frontal sinuses showing two calvarial fragments were displaced with the main one being depressed into the lumen of the left frontal sinus (white arrow). **c** Three-dimensional reconstructed volume rendering of the skull showing a right frontal depressed bone fracture

A right-sided frontal bone depression fracture was suspected founded on clinical findings. Radiographs and Computer Tomography (CT) imaging with a three-dimensional reconstruction of the skull were performed under general anaesthesia (see Fig. 1a-c). Radiographs of the cervical spine were unremarkable.

CT imaging revealed a comminuted, depressed fracture of the frontal bone that extended from the level of the maxillary recesses up to the caudal aspect of the frontal sinuses at the level of the dorsal aspect of the right maxillary, nasal and frontal bones. Surgical repair of the defect was warranted to reestablish sinus architecture and mechanical stability [10]. Further, fracture comminution is associated with soft tissue contracture leading to cavitation with connective tissue scarring and sequestrum formation [14], fracture repair addresses soft tissue injury and may minimize long-term risks of complication [15]. Surgery was carried out three days after admission.

**Fig. 2:**
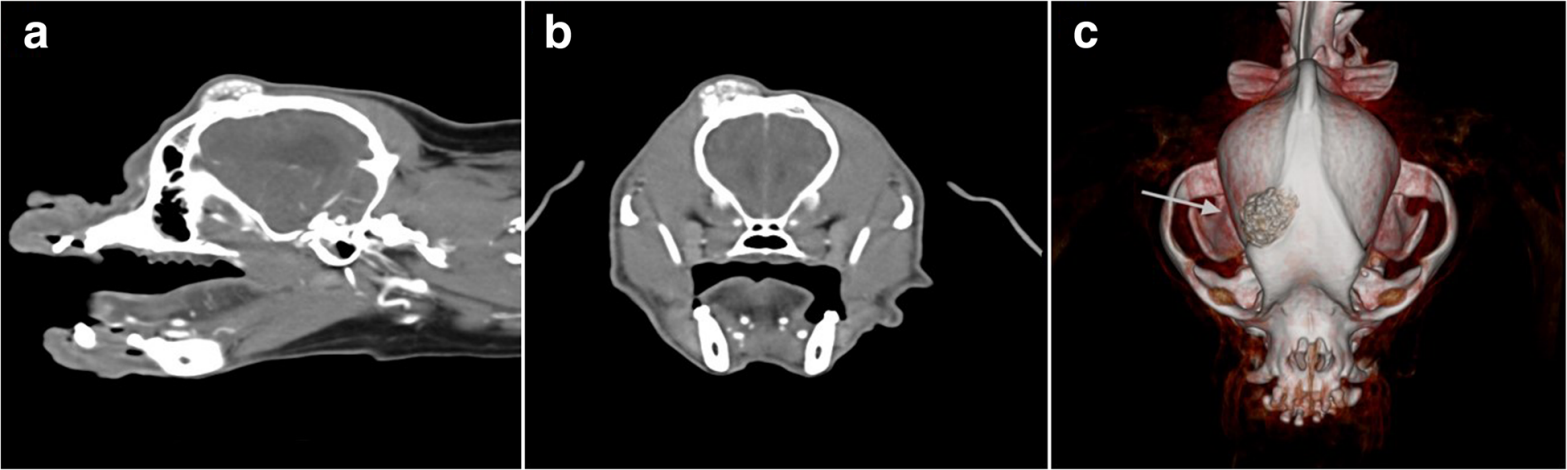
**a** Parasagittal CT scan reconstruction showing a 25mm height x 20mm diameter ovoid mass arising from the right frontal bone above the right orbital globe. **b** Transverse CT scan image of the skull showing the mass arising from the right frontal bone. **c** Three-dimensional reconstructed volume rendering of the skull showing the mass arising from the right frontal bone (white arrow).

A standard dorsal approach to the frontal bone was taken (Fig. 2-a). A malleable highly porous Ti mesh (0.2 mm thickness with 1.4 mm by 0.6 mm elongated pores) was contoured to the patient’s skull (Fig. 2-b).

The Ti mesh was firmly seated against the skull and a 1.6 mm drill bit, with a 6 mm stop, was used to drill pilot holes through the mesh into the skull. A 2.1 mm diameter resorbable poly-L, D-lactic acid (PLDLA) thermoplastic pin was placed into the pilot hole and the ultrasonic trode applied to the proximal end of the pin (Fig. 2-c). The ultrasonic device was activated melting the outer surface of the pin and allowing it to be advanced into the pilot hole. Further pilot holes were drilled around the periphery of the Ti mesh at 20-25 mm intervals and thermoplastic pins inserted until the Ti mesh was adequately secured.

**Fig. 3:**
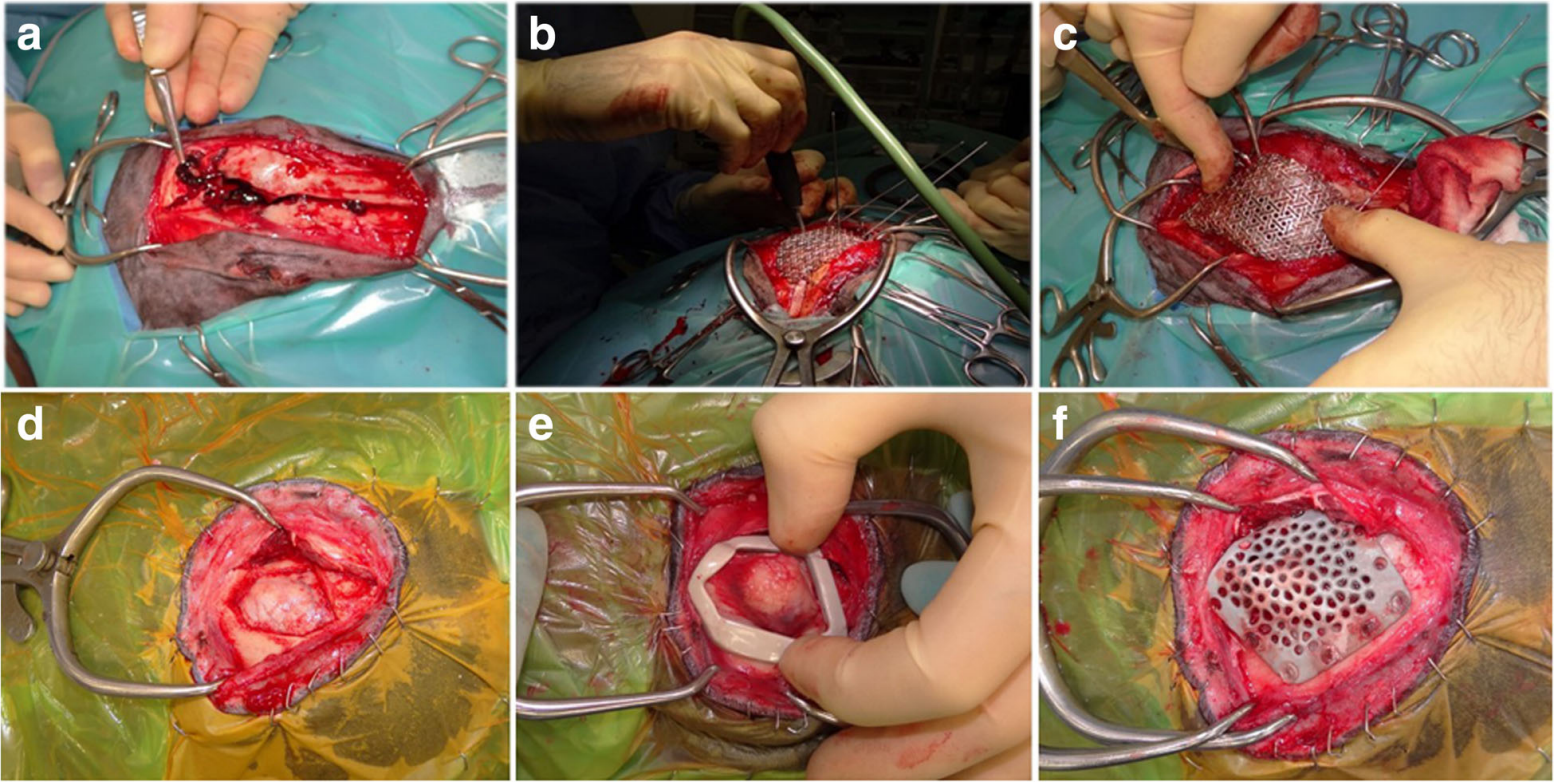
**a** Surgical exposure of the calvarial fracture site. **b** Intraoperative use of the ultrasonic device and **c** Intraoperative contouring of the titanium mesh (Case 1). **d** Surgical exposure of the calvarial resection site. E) Custom template designed for border demarcation and (F) Titanium mesh secured with nonresorbable polymer pins covering the defect (Case 2)

The surgical site was flushed with copious volumes of sterile saline and a sheet of gentamycin-impregnated collagen (Collatamp®, EusaPharma) was overlaid on the implant to prevent infection and assist in achieving a pneumatic seal. Prophylactic intravenous antibiotic cover was provided by clavulanated amoxicillin (20 mg/kg; Augmentin, GSK) given 30 min pre-operatively and every 90 min thereafter for surgery duration. Recovery from anaesthesia was uneventful. Post-operative analgesia was provided with intravenous methadone (0.2 mg/kg q4hours; Comfortan, Dechra). Postoperative radiographs and CT images showed satisfactory positioning of the Ti mesh and adequate coverage of the calvarial defect (Fig. 3-a, b, c).

The dog was discharged two days after surgery with a 10 day course of oral carprofen (2 mg/kg q24hours; Rimadyl, Zoetis) and oral clavulanic acid-amoxicillin (20 mg/kg q12h; Synulox, Zoetis).

The dog re-presented two weeks after discharge, with hyperthermia and swelling around the surgical site. Subcutaneous oedema was present in the absence of emphysema. Sampling for culture and sensitivity was prevented by the absence of nasal and surgical site discharge, exposure of the wound to obtain a swab sample was deemed inappropriate. Antibiotic therapy effective against common nasal pathogens [16] was introduced with oral cefalexin (20 mg/kg q 12 hours; Therios, Ceva) for ten days. Clinical signs had resolved at 1 week post treatment.

**Fig. 4:**
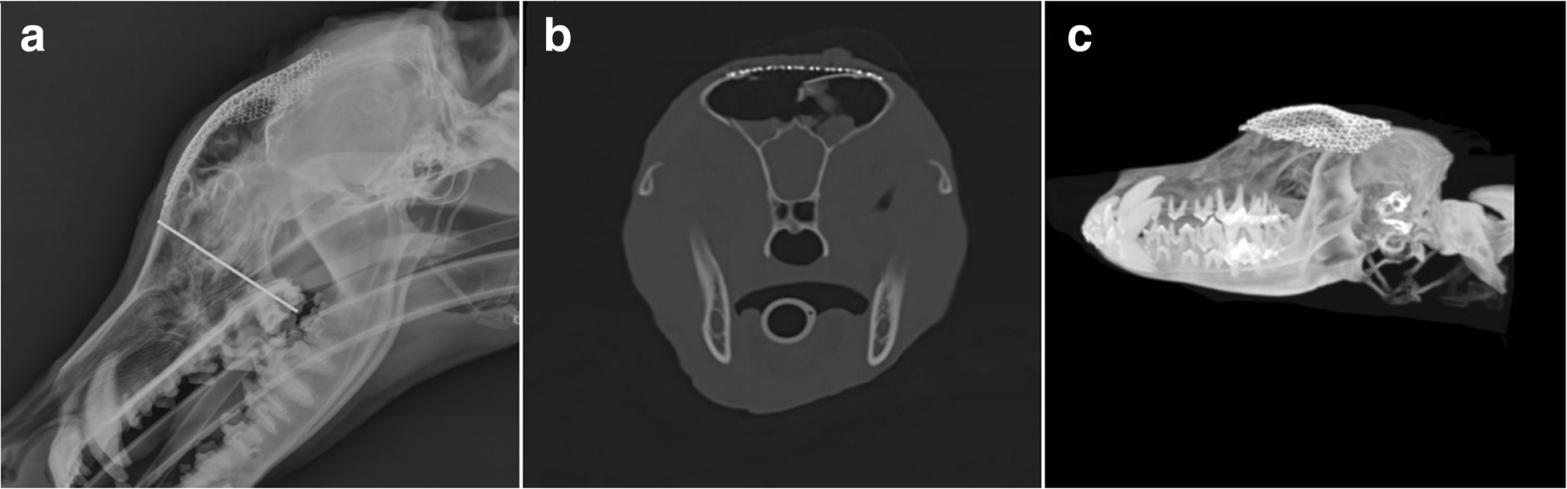
**a** Lateral immediate postoperative radiographic view of the skull showing titanium mesh spanning from the level of the third premolar maxillary tooth (grey line) until the level of the parietal bones. **b** Immediate postoperative transverse CT reconstruction at the level of the frontal sinuses showing a titanium mesh repair spanning the right frontal bone fracture. **c** Three-dimensional reconstruction of the skull showing the titanium mesh covering the caudal aspect of the maxillary bones and rostral frontal bone

At the six weeks recheck, the physical examination was within normal limits. No pain was elicited upon palpation of the surgical site and no indications of infection recurrence was found. Radiographs and CT of the skull revealed a slight uplift of the mesh at its most rostral aspect from the frontal bone. Mesh uplift was not a clinical concern at this stage. Radiographs and CT scan at 6 months revealed soft tissue swelling between the rostral mesh portion and the skull (see Fig. 4-a). Decision was made to trim and re-contour the uplifted mesh. The procedure and recovery were uneventful and no further complications were experienced.

At 2 years assessment, radiographs and CT scan confirmed adequate contouring and positioning of the Ti mesh. Radiographs revealed a bone-dense opacity between the rostral part of the mesh and the skull potentially associated with mesh micromotion stimulating periosteal reaction (Fig. 4-b). The degree of new bone formation around the mesh periphery was difficult to ascertain radiographically, however, clinical examination was unremarkable and cosmetic result was satisfactory. The owners did not raise any further concerns regarding the patients ability to participate in normal lifestyle and activity.

### Case 2

**Fig. 5:**
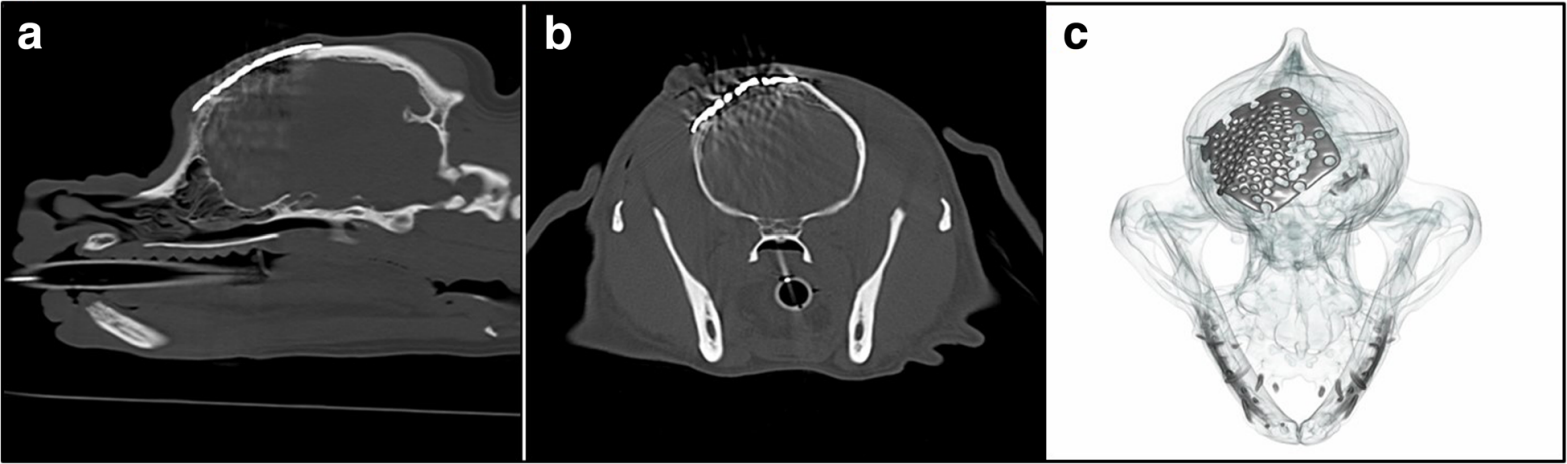
**a** Immediate postoperative sagittal CT scan reconstruction of the skull showing adequate covering of the surgical defect in the right frontal bone by titanium mesh. **b** Immediate postoperative transverse CT scan reconstruction. **c** Three-dimensional volume rendering reconstruction of the skull with mesh in situ

A ten-year-old neutered female Cavalier King Charles Spaniel presented for a gradually enlarging mass on the right frontal bone with no associated clinical signs. The mass was bone-like and non-painful upon palpation. The remainder of clinical examination was within normal limits. Fine needle aspirates of the mass revealed evidence of bone remodeling compatible with a neoplastic process yet were not diagnostic, further investigation was declined and mass excision by surgery was planned. Radiography and CT imaging of the skull were performed under general anaesthesia to advise surgical planning and custom 3D Ti mesh design for use in reconstruction following tumour resection. Thoracic and abdominal CT scan were also taken for staging and were negative for metastatic disease. CT imaging of the skull revealed a 25 mm (h) × 20 mm (diam.) ovoid mass arising from the right frontal bone above the right orbital globe (see Fig. 5a-c). Surgery was implemented a week later.

The same surgical approach was performed (Fig. 2-d). A craniotomy with resection margins guided by the use of customised template was performed (Fig. 2-e). The resection guide was computer modelled from CT imaging and printed using a sterolithographic system. A pre-contoured Ti mesh aided by computer modeling and computed manufacture was firmly seated to the patient’s skull covering the defect (see Fig. 2-f). The protocol for thermoplastic pin placement and application of gentamycin-impregnated collagen sheet was as described for patient 1. Non-resorbable methacrylate-butadiene-styrol (MBS) pins were used in case 2 for fixation in contrast to resorbable PLDLA described in case 1.

**Fig. 6:**
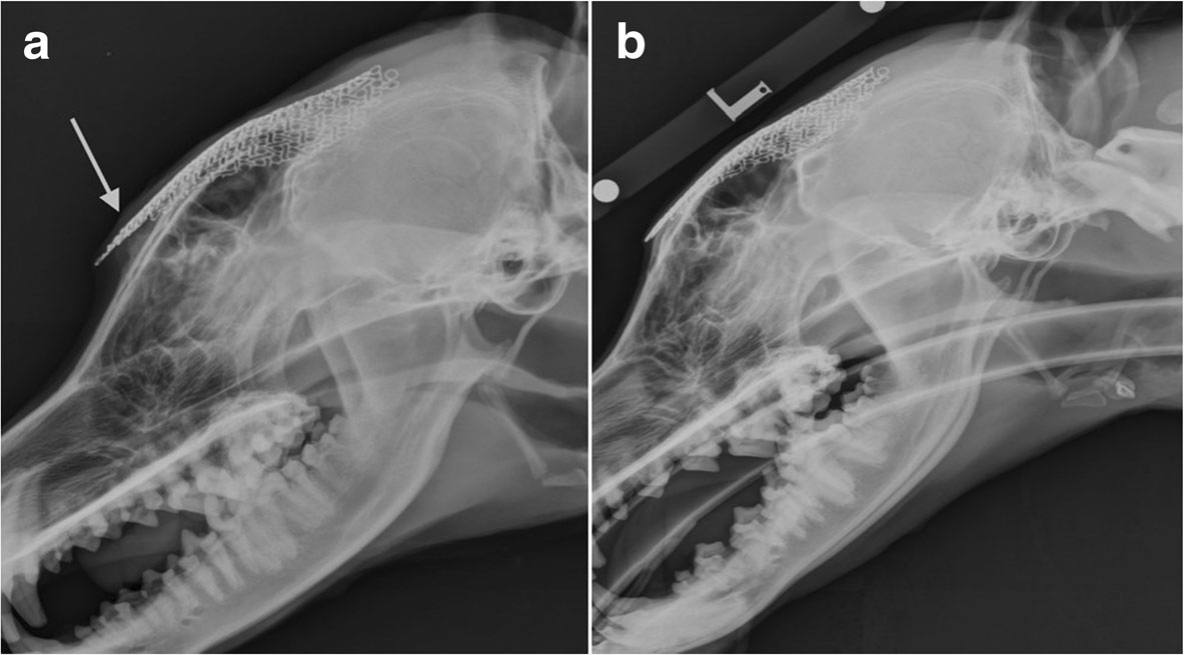
**a** 6 months postoperative lateral radiographic view of the skull showing rostral uplift of the mesh (white arrow). **b** 2 years postoperative lateral radiographic view of the skull showing adequate positioning of the mesh

Prophylactic intravenous antibiotic cover was provided by cefuroxime (20 mg/kg; Zinacef, GSK) given 30 min pre-operatively and every 90 min thereafter for surgery duration. Recovery from anaesthesia was uneventful. Post-operative analgesia was provided with intravenous methadone (0.2 mg/kg q 4 h) and in travenous paracetamol (10 mg/kg q12hours; Perfalgan, BristolMyers squibb). Postoperative radiographs and CT images showed satisfactory positioning of the Ti mesh and adequate coverage of the calvarial defect (Fig. 6-a, b, c). The dog was discharged six days after surgery after uneventful hospitalisation.

The dog re-presented two weeks after discharge for suture removal. Surgical wound has healed and physical examination was within normal limits. At the ten weeks recheck, physical examination was within normal limits and the owner reporting no restrictions on the patient’s activity. There was no pain elicited upon surgical site palpation. Radiographs and CT of the skull revealed satisfactory positioning of the mesh. A histological diagnosis of multilobular osteochondrosarcoma was made with potential of local recurrence advised.

At 7 months assessment, owners reported a small bone-like mass near the resection site. This clinical finding was consistent with either local recurrence or reactive osseus proliferation. CT scan identified the region of proliferation as dorsal and to the right side of the Ti mesh. The mesh remained well localised and confluent with the skull. The remaining clinical examination was unremarkable and cosmetic result was satisfactory. Owners did not raise concerns regarding overall quality of life and further investigation of the mass was declined. A request to examine the patient at 1 year follow up was again declined by the owner due to long travel distance.

## Discussion

Three main goals should be achieved when reconstructive maxillofacial surgery is performed: biomechanical stability, cerebral protection and cosmetic restoration [17, 18]. In case 1, medical management was not deemed appropriate as the scale of damage and exposure of nasal sinus was considered significant enough to undermine mechanical integrity of the facial and skull bones. Referenced surgical treatment modalities of maxillofacial fractures in humans advocates sinus reconstruction and restoration of mechanical stability for the protection of sensitive intracranial structures and limitation of infection risk [10]. Although specific guidelines pertaining to canine surgery could not be found it was the authors’ clinical decision that guidelines for humans be consistent with the clinical need in case 1, relating to similarity of intracranial structure and infection risk. Of importance was the possibility for infection originating from sinus flora, advanced sequencing of nasal canine flora highlights a number of potentially pathogenic bacteria present in healthy dogs [19], hence the potential for infection as a consequence of a compromised sinus architecture supports the require of surgical management in this scenario.

Materials used for skull defect reconstruction can include autologous bone, allogenic and alloplastic materials, synthetic and organic biomaterials, or biocompatible metals and their alloys. Titanium mesh is used in human surgery for its strength, malleability, ability for easy intraoperative handling, biocompatibility and low infection rate [20–23]. These systems are routinely used for defect reconstruction in maxillofacial surgery for the treatment of depressed skull fractures and reconstruction of paranasal sinuses. Application of Ti mesh within veterinary medicine is less widespread but is documented in skull reconstruction following primary bone tumour removal [2, 5].

In case 2, the Ti mesh was placed over an intact dura after tumor resection. In the event of compromise to the dura, augmentation of the dural layer facility is indicated as previously described [5]. Dural repair materials should be biocompatible, non-toxic and may include autologous substances (pericranium, temporalis fascia and fascia lata) or biological substitute materials such as Small Intestinal Submucosa [20].

Titanium mesh provides long-term biomechanical stability [23] and may be used as a framework for soft tissue reconstruction over the damaged sinus. In the current cases, a complete air-tight sealing of the sinuses was obtained with the combination of Ti mesh, gentamycin-collagen sponge and soft tissue closure after accurate reconstruction and remained unchanged in the long-term follow-up (up to two years).

The innovative use of polymeric pins with Bonewelding® technology may carry several advantages compared to traditional metallic screws and plates. Biocompatibility of the polymer screw technology was demonstrated in an ovine spine fusion model. The system demonstrated bone-screw integration, high bonding strength when assessed by screw pull-out and clear new bone growth surrounding the implant [24, 25]. Application of Bonewelding® is considered less traumatic than traditional metallic screw fixation [26] and is advantageous in cases where the skull defect originated from trauma, minimising further inflammation whilst achieving surgical repair and implant integration. In case 1, the calvarial defect originated from trauma precipitating severe localised tissue inflammation, the decision to use Bonewelding ® technology was in part based on an intention to minimise further damage to traumatised tissue.

In an advantage, pins are not constrained to pre-determined trajectory as defined by implant design and threading; the angle of application can be varied without undue application of torque forces [25]. This ease of use improves the procedural flexibility lending itself to use in the repair of irregular bone defects and in clinical scenarios involving areas of complex morphology. In the reported two cases, the location and complex geometry of bone defects benefitted from the procedural flexibility afforded by employing Bonewelding® technology. The unusual nature of the reported cases negates the possibility of comparing surgical time to standard fixation and comparable reported procedures do not document surgical duration [5]. Application of the polymer pin system was perceived as being faster compared to conventional screws, as it has been reported by Pilling et al. [25] and Eckelt et al. [12]. However, categorical demonstration of this fact in this instance was not possible.

Polymer pins are radiolucent and therefore have an advantage for postoperative CT studies compared to metal implants generating fewer artefacts allowing more accurate assessment of the operated site [13, 27]. Quantification of postoperative bone formation and mesh-skull integration was complicated although satisfactory mesh positioning at long-term follow up may be interpreted as an adequate clinical indicator of tissue incorporation. The authors thought it warranted to use both radiographic and CT scan modalities as diagnostic tools thoroughly reflect of the postoperative appearance of this novel polymeric fixation material. Although CT scan is the technique of choice to assess such an implant, radiography remains the most widely available and easy to perform modality in first opinion practice and as such the accurate description of the radiographic presentation of the material carries direct clinical relevance.

Complications associated with the use Ti mesh for skull defect reconstruction in humans include infection, haemorrhage, seizures and postoperative pain [27, 28]. In previous veterinary reports, postoperative complications after Ti mesh cranioplasty have not been described [2, 5, 29].

In case 1, the mesh uplift in its rostral aspect six weeks postoperatively was an incidental finding on imaging. It is the authors’ opinion that the irregular rostral border of the mesh implant may have required further fixation to the rostral frontal and caudal maxillary bones. It was intended that accurate contouring of the mesh in combination with pin placement would have secured the mesh sufficiently. Melting and fusion of the polymer pins in the host bone by ultrasound increase the mechanical resistance to shearing forces compared to a conventional screw-plate construct [30] and provide sufficient mesh fixation. Increased pin number and density may circumvent complications of inadequate implant constraint although conjecture remains regarding the suitability of a resorbable system in the reported case. In case 2, non-resorbable pins made of an acryl-based multipolymer compound, MBS, were used and despite the presence of an osseous response which could not be investigated, mesh positioning remained well seated to the defect and no implant related complications were experienced.

Use of polymeric pins poses challenges to the new user. Attention must be given to the angle of pin placement and degree of ultrasonic exposure received. Incomplete insertion can occur if ultrasound application is removed too early [31]. Similarly, if the process of pin introduction during ultrasound application is excessively delayed, it can impair proper fusion and result in inadequate linkage of the pin-bone interface [32]. Resorbable polymers may be contraindicated where high mechanical load or movement occur as their degradation may occur before new bone formation, leading to implant loosening [31]. Bonewelding® resorbable pins were made of PLDLA and resorb within approximately 10–12 weeks. The degradation profile of PLDLA pins was designed to facilitate tissue in-growth consistent with rate of polymer absorption [33].

Ideally, the breakdown, resorption and excretion of a resorbable system should coincide with new tissue growth and ease transition from synthetic fixation to bony integration with the implant. The breakdown of PLDLA in vivo occurs by hydrolysis, subsequent phagocytosis and cyclic breakdown into lactic acid. Initial hydrolysis reduces polymer molecular weight without affecting the total bulk (and by extension strength). As the PLDLA is further cleaved, the number of polymer chains exposed for hydrolytic and enzymatic degradation increases, accelerating the rate of degradation. Polymer breakdown is influenced by implant shape, size, molecular weight, pH and the water content of the surrounding tissue (contributing to hydrolysis) [34]. Polymer breakdown products can influence implant microenvironment stimulating an inflammatory response and, although documented in humans, reaction to PLDLA is uncommon in animal models [35]. In a clinical setting, factors influencing tissue repair and new bone growth may result in a disconnect between polymer breakdown and the intended transition towards biological fixation. In this scenario the implant would no longer be adequately secured and migration related complications would be expected. Mesh stabilization was better maintained in the patient wherein non-degradable pins were used for fixation. The authors would propose that the uplift of mesh in case 1, which appears concurrent with the expected timing of pin degradation, and the degradable poly-pin can be causally linked. In case 2, the absence of mesh uplift may be linked to the use of non-resorbable pins although no further consensus is possible with larger and extensive case series.

A gentamycin-collagen sponge was used to deliver high-concentration perioperative antibiotics to reduce risks of infection [36]. Collagen has been used as a natural polymer scaffold for applied growth factors, cytokines and transplanted cells which may enhance osseointegration and bone repair [37].

Osseointegration at the mesh-bone interface could have been achieved through traditional autograft or allograft or synthetic materials. The use of octacalcium phosphate combined with collagen disks enhanced bone regeneration up to six months after application in a canine calvarial defect suggesting a potential role as a bone substitute graft material [38]. The application of exogenous Bone Morphogenic Protein 2 by a polymer delivery system significantly increased bone formation compared to collagen implant alone in calvarial defect model in rats [39]. Pro-osteogenic material coatings such as HA enhance osteoblastic activity and increase mesh osteoconductivity [40].

## Conclusions

We report the successful repair of a traumatic calvarial fracture in a greyhound and calvarioplasty in a Cavalier King Charles Spaniel using a Ti mesh secured with thermoresponsive polymer pins. It is in the authors’ opinion that Bonewelding ® technology may be a suitable adjunct technique for fixation of calvarial defects as it yields easy application with minor bone trauma. The short-term postoperative mesh uplift encountered in case 1 with the use of resorbable pins was a potential consequence of their variable degree of degradation and incomplete biological integration, comparatively no complications relating to implant movement were reported with the use of the non-resorbable system in case 2, post-operatively and at 7 month follow up. The varying outcome using absorbable and non-absorbable systems may suggest a clinical advantage to the non- absorbable pins, however a multiple patient case series with long term follow up would be required to demonstrate this proposal.

## Abbreviations

CT: Computer tomography
HA: Hydroxyapatite
MBS: Methacrylate-butadiene-styrol
PMMA: Polymethylmethacrylate
Ti: Titanium
